# Therapeutic effects of sequential therapy by electric coagulation, cryotherapy and balloon dilation with an electronic video bronchoscope

**DOI:** 10.3892/etm.2013.1031

**Published:** 2013-03-29

**Authors:** EN-QING FU, YAN-DONG NAN, FA-GUANG JIN, AI-QUN MA

**Affiliations:** 1 Department of Cardiovascular Medicine, The First Affiliated Hospital, Medical College of Xi’an Jiaotong University, Xi’an, Shaanxi 710061;; 2 Department of Respiratory Medicine, Tangdu Hospital, The Fourth Military Medical University, Xi’an, Shaanxi 710038, P.R. China

**Keywords:** coagulation, cryotherapy, balloon dilation, bronchostenosis, bronchoscopy

## Abstract

The aim of the current study was to retrospectively analyze clinical data concerning bronchostenosis or bronchial obstruction caused by endobronchial tuberculosis. Fifty-six cases were subjected to bronchoscopy and chest computed tomography to assess the prognosis of bronchostenosis and bronchial obstruction. Based on reliable and effective anti-pulmonary tuberculosis therapy, these conditions were treated sequentially by electric coagulation, cryotherapy and balloon dilation with an electronic video bronchoscope during outpatient consultation or inpatient hospitalization. Fifty-three subjects with bronchostenosis recovered to varying degrees, a recovery rate of 94.6%. Thirteen of the 15 cases with bronchial obstruction reopened (86.7%). The clinical symptoms of these cases appeared to be in remission. Bronchostenosis or bronchial obstruction resulting from endobronchial tuberculosis may be treated by electric coagulation, cryotherapy and balloon dilation with an electronic video bronchoscope.

## Introduction

The incidence of pulmonary tuberculosis (PTB) has increased globally, particularly in developing countries. Injuries from PTB have worsened, causing more complex therapeutic difficulties. The mortality rate for PTB has continued to rise. Clinically, severe bronchostenosis or bronchial obstruction of the trachea, main bronchus and lobar bronchi often occur prior to, during and following the treatment of endobronchial tuberculosis. The accompanying lesions may result in atelectasis of the left lung, right lung or lung lobes, which limits the efficacy of the anti-PTB therapy. Thus, certain PTB patients suffer from clinical symptoms, including severe chest distress, shortness of breath and dyspnea. Medication is unsuccessful for these patients and pulmonary function fails to recover following surgery. Pulmonary lobectomy of the lung or the lung lobes, performed when the medical condition is stable, often has severe negative consequences in patients (1). At the Respiratory Department of Tangdu Hospital, The Forth Military Medical University (Xi’an, China), reliable and effective anti-PTB therapy has been combined with sequential therapy through electric coagulation, cryotherapy (2) and balloon dilation (3–9) using an electronic video bronchoscope to treat patients with bronchostenosis or bronchial obstruction without stenting (10–14). This technique yielded encouraging results prior to, during and following the treatment of endobronchial tuberculosis. The technique primarily eliminated the bronchostenosis or bronchial obstruction, healed atelectasis and preserved the pulmonary function with satisfactory therapeutic effects. Our complex method varies slightly from previously reported methods (15,16).

## Patients and methods

### 

#### Clinical data

A total of 56 subjects with bronchostenosis or bronchial obstruction caused by endobronchial tuberculosis were selected for this study. These subjects were treated sequentially with electric coagulation, cryotherapy and balloon dilation using an electronic video bronchoscope. The procedures were performed during outpatient consultation or inpatient hospitalization in the Department of Cardiovascular Medicine, First Affiliated Hospital, Medical College of Xi’an Jiaotong University, China. The subjects were treated from January 2007 to December 2009. The recovery of the patients was monitored for a year. The 56 patients consisted of 21 males and 35 females, aged 5–56 years (mean, 26.8±12.5 years). The diagnosis of endobronchial tuberculosis was confirmed by biopsy pathology with an electronic video bronchoscope (model 1T-240 or -260, Olympus, Tokyo, Japan) or by the detection of *tubercle bacillus* in the patient’s sputum aided by the tuberculin test (PPD test). The stenosis sites in the bronchi or lobar bronchi are specified in Table I. All patients provided informed consent. The treatment method was verified by the Ethics Committee of Tangdu Hospital and the procedure was approved. Five of the 56 patients completed the entire course of anti-PTB therapy and then the use of anti-PTB drugs was stopped. These patients only exhibited bronchostenosis, which was later corrected through balloon dilation. The other 51 patients continued with the anti-PTB treatment. Several patients presented poor treatment efficacy when evaluated via electronic video bronchoscopy. These patients were then subjected to a modified and reinforced therapy and the therapeutic effects were positive. All 56 cases received the tuberculin test and the results are presented in Table II. The surgery staff wore N97 masks for self-protection and no evidence of cross-infection was observed.

#### Treatment with anti-PTB medicine

All patients received a combination of anti-PTB drugs. The appropriate anti-PTB drug combination (0.3 g isoniazid, 0.45 g rifampicin, 0.75 g ethambutol and 0.5 g pyrazinamide taken three times daily; a dose increase or reduction was made for several patients) switched to 2HRZE/9HR after two months (0.3 g isoniazid and 0.45 g rifampicin). This was administered under constant observation during the entire treatment period. Patients with endobronchial erosion received isoniazid and dexamethasone through ultrasonic gas-atomization technology. Patients who were resistant to the anti-PTB drug combination were provided with second-line anti-tuberculosis drugs [ethionamide, physiological saline (PS), *para*-amino-salicylate and levofloxacin].

#### Microtrauma and video bronchoscopy

The topical treatments included sequential electric coagulation with an argon plasma coagulator (ARCO-3000; Söring GmbH, Quickborn, Germany), cryotherapy using a cryotherapy machine (Dräger, Lübeck, Germany) and dilation using high-pressure balloons of different diameters (Johnson & Johnson, New Brunswick, NJ, USA).

#### Electric coagulation

The main granulation tissue that was not frozen and eliminated was selected and the tissue was coagulated using the argon plasma coagulator until it carbonized. The process was repeated several times.

#### Cryotherapy

The main necrotic tissues that it was possible to freeze and eliminate by cryotherapy were selected, so according to the ice ball mechanism, the tissues would be swollen. The ice ball is formed in the cryotherapy detection head at the beginning of cryotherapy. The freezing detecting head was placed in one point for one to two minutes each time. A section with bronchostenosis was treated repeatedly several times. The treatment was normally administered once a week.

#### Balloon dilation

Numerous methods of balloon dilation exist (4–9). The majority of methods involve continuous dilation for 2 min and the highest pressure of dilation is <2 atm. Our method is entirely different from these methods. Our novel high-pressure balloon dilation method includes the following steps: insertion of the video bronchoscope, insertion of the guide wire along the gravitational duct of the bronchoscope, removal of the bronchoscope, reinsertion of the bronchoscope, insertion of the high-handed balloon along the guide wire, injection of physiological saline through the balloon duct with a booster pump to increase the pressure in the balloon (the pressure of balloon increased from 0 to 14 atm) and removal of the video bronchoscope. The patient was seated for 40 min for continuous dilation. Then, the bronchoscope was inserted again and the balloon and guide wire were removed following observation of the bronchus. Finally, the bronchoscope was removed. Balloon dilation was not performed if the diameter of the bronchial stenosis was greater than two-thirds of the diameter of the normal bronchus.

#### Sequential therapy for bronchostenosis with a video bronchoscope

Sequential therapy was performed. A topical anesthetic spray with 2% tetracaine and a 2% lidocaine drip were administered through a bronchoscope instead of general anesthesia. For patients with bronchostenosis caused by endobronchial ‘cheesy’ necrosis, the necrotic tissues were frozen and excised and the bronchial wall was frozen. The procedure was repeated until the intima healed and became smooth. For patients with endobronchial ‘cheesy’ necrosis accompanied by bleeding, the necrotic tissues were refrigerated and excised once the bleeding had stopped using the argon plasma coagulator and the bronchial wall was treated through cryotherapy until the intima became smooth. In cases where bronchostenosis remained following these procedures, a high-pressure balloon was utilized (pressure, 4–12 kPa) to dilate the inner diameter of the bronchus to recover its pulmonary function and to relieve symptoms, including dyspnea. A 64-row computed tomography (CT) examination for bronchial imaging was performed on patients with bronchial obstruction to assess whether the distant bronchus was obstructed. In cases where the bronchus was open, the site of stenosis was treated with cryotherapy to reduce the size of the closure. Then, the closure was reopened using the argon plasma coagulator. When the opening became larger, cryotherapy was performed until the intima became smooth, followed by the balloon dilation method (5,17,18) to maximize the recovery of the inner bronchus. If the distant bronchus is obstructed, the above mentioned procedure is not applicable. Normal lung inflation, as well as lung function, was gradually restored when medications were administered.

#### Timing of bronchoscopic examination and treatment

The first examination was conducted on patients upon admittance to hospital and the first video bronchoscope treatment followed as necessary. The treatment was based on the damaged tissue of the local bronchi. The succeeding treatments were administered once a week until the damage was alleviated. Then, the treatment was provided once every two weeks or once a month. When the damaged focus disappeared completely, the patients received their last examination and were asked to stop taking all the anti-tuberculosis medicine.

#### Standard effect of bronchostenosis

The diameter of the bronchus before and after bronchoscopic balloon dilation and before the patient was cured of PTB was measured. Then, the patients were asked to stop taking all treatments (∼18 months after treatment). The diameters of the bronchoscopes were considered as the control values. The diameter of the Olympus BF260 video bronchoscope was 4.9 mm and of the Olympus 1T260 bronchoscope was 5.9 mm. The diameters of the bronchi were measured, recorded and compared with the diameter of the bronchoscopes.

#### Standard effect of bronchostenosis on bronchial diameter

A bronchial diameter that was one-third to two-thirds the size of a normal bronchus following treatment indicated that the treatment was effective. The diameter of the cured bronchus was determined and compared with the diameter of the normal bronchus. If the diameter of the cured bronchus was less than one-third of the diameter of the normal bronchus, then the treatment was not effective.

## Results

### 

#### Clinical effect

Fifty-three of the 56 cases (94.6%) of bronchostenosis recovered to varying degrees. The mucous membranes healed and became smooth. Symptoms, including chest distress, shortness of breath and dyspnea, improved. Thirteen of the 15 cases (86.9%) of bronchial obstruction reopened. The majority of the pulmonary atelectatic tissues were dilated. The overall rate of recovery for all cases was 90.4%. In two of the cases, it was not possible to reopen the long segment of bronchial obstruction and dilate the pulmonary tissues distal to the obstruction; thus, these two patients discontinued the treatment. The patients underwent cryotherapy 20 times in a period of six months and had five sessions of balloon dilation within the same time period. The high-pressure balloons were selected in a sequence according to the inner diameter of the bronchus with bronchostenosis or bronchial obstruction (3–15 mm) during therapy. The opening of the bronchus was dilated as much as possible to allow the inner diameter of the bronchus to return to its normal size.

### Typical cases

#### Case 1

**Figure 1:**
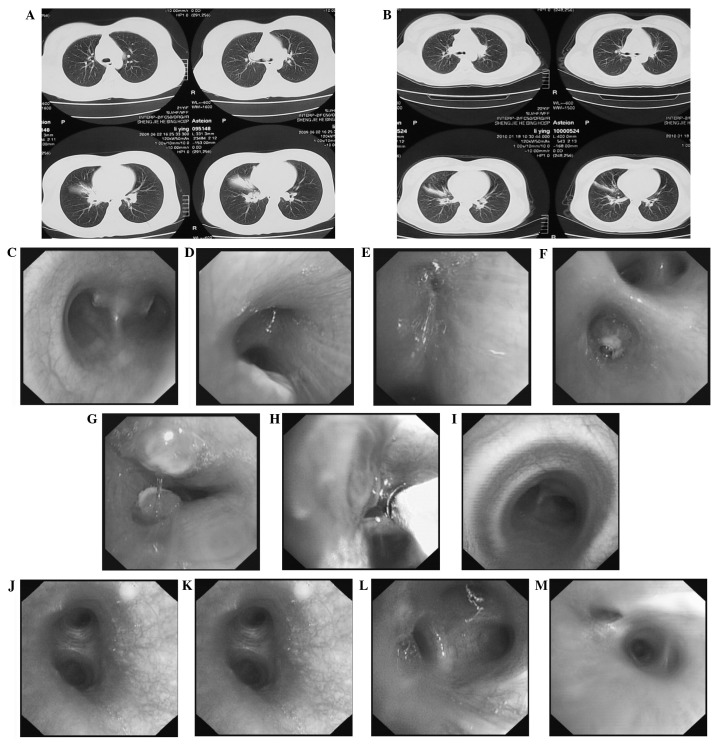
Case 1. (A). Computed tomography (CT) imaging prior to the cryotherapy procedure (7/3/2009); (B) CT imaging following the cryotherapy procedure (1/10/2010). Bronchoscopic images prior to treatment (7/27/2009) in the (C) carina; (D) left main bronchus; (E) upper lobe of the right lung; (F) middle and lower lobes of the right lung; (G) lower lobe of the left lung and (H) during cryotherapy. Bronchoscopic images following treatment (4/6/2010) in the (I) carina; (J) left main bronchus; (K) upper lobe of the right lung; (L) middle and lower lobes of the right lung and (M) lower lobe of the left lung.

A 22-year-old female was diagnosed on March 4, 2009 with double PTB and bronchial tuberculosis marked by a month of coughing, expectoration, chest distress and shortness of breath. Anti-PTB treatment (isoniazid, rifampicin, ethambutol and pyrazinamide) was administered; however, it produced poor results. Through an electronic video bronchoscope, ‘cheesy’ necrosis was noted in the carina, right main bronchus, end of the left main bronchus, opening of the upper, middle and lower lobe of the right lung and the opening of the left lower lobe, which obstructed the trachea. Combined with a treatment of isoniazid (0.1 g) and sodium chloride (20 ml) and through the ultrasonic gas-atomization technology, cryotherapy was conducted 19 times through an electronic video bronchoscope. The patient’s condition improved. Fig. 1A and B show CT images before and after cryotherapy, respectively. Fig. 1C–G, Fig. 1H and Fig. 1I–M show bronchoscopic images before, during and after cryotherapy, respectively.

#### Case 2

**Figure 2:**
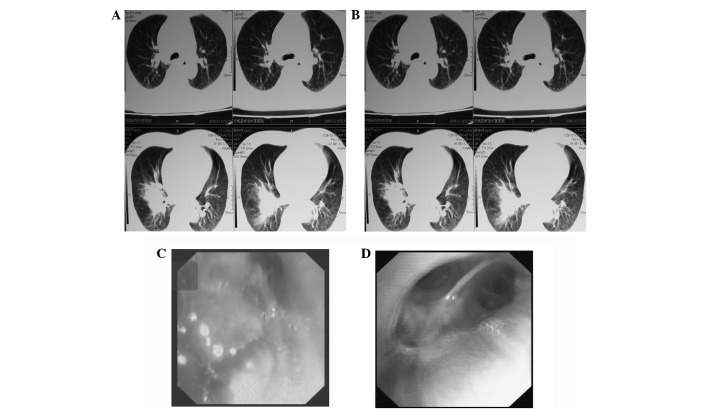
Case 2. (A) Computed tomography (CT) imaging prior to treatment (3/2/2009); (B) CT imaging following treatment (10/20/2010); (C) upper lobe of the right lung prior to treatment (3/11/2009); (D) upper lobe of the right lung following treatment (10/20/2010).

A 43-year-old male was diagnosed on March 10, 2009 with PTB in the right upper lobe and bronchial tuberculosis marked by two months of coughing and expectoration. The patient underwent treatment in another hospital for three months; however, the treatment was ineffective. Through an electronic video bronchoscope, granulation tissue covering the right upper lobar bronchus was noted, which obstructed the majority of the right upper lobe. Through the combination of isoniazid (0.1 g), sodium chloride (20 ml), ultrasonic gas-atomization technology (twice a day for two months) and anti-PTB therapy (isoniazid, rifampicin, ethambutol and pyrazinamide), cryotherapy with an electronic video bronchoscope was conducted 17 times. The patient’s condition improved. Fig. 2A and B present CT images before and after cryotherapy, respectively. Fig. 2C and D present bronchoscopic images before and after treatment, respectively.

#### Case 3

**Figure 3:**
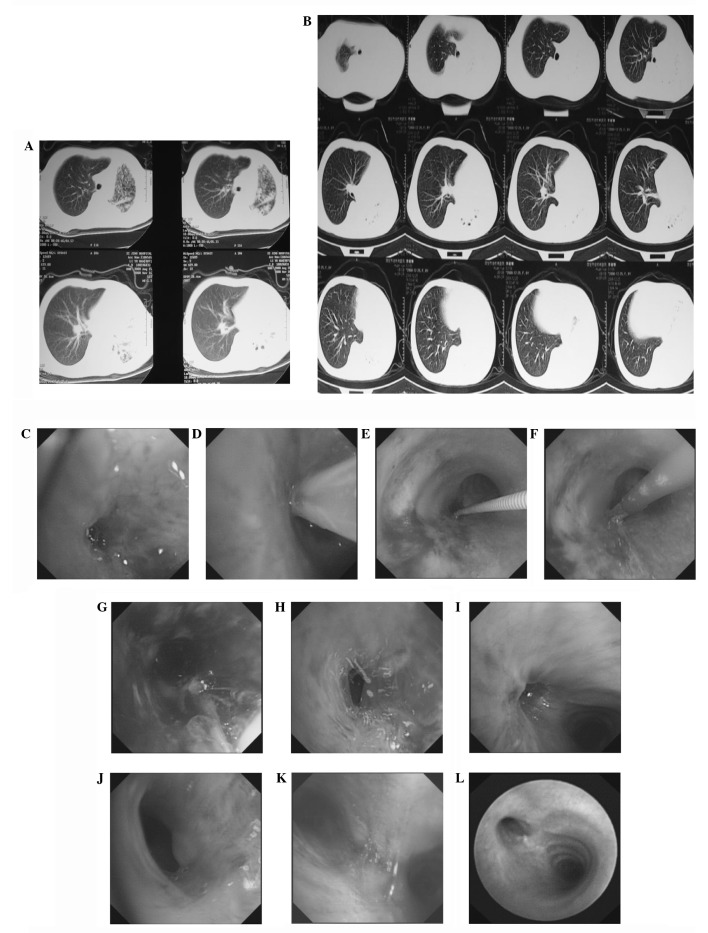

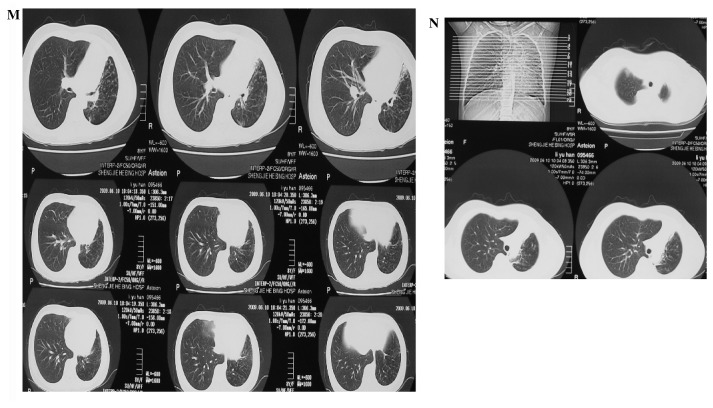
(A) Computed tomography (CT) imaging 2 weeks before treatment (03/01/2009); (B) CT imaging following anti-pulmonary tuberculosis (PTB) therapy (03/19/2009); (C) left main bronchus; (D) argon plasma coagulation; (E) insertion of guide wire; (F) prior to first balloon dilation; (G) following first balloon dilation; (H) rechecked bronchus partial coarctation; (I) second balloon dilation; (J) fourth rechecking; (K) third balloon dilation. (L) Left main bronchus was unobstructed with partial bronchostenosis. (M and N) computed tomography (CT) imaging following treatment (06/10/2009).

A shadow in the left lung of an 8-year-old female was detected by chest CT. The patient reported two months of coughing and shortness of breath and was diagnosed with left lung tuberculosis and endobronchial tuberculosis based on the discovery of tubercle bacillus in the sputum. The patient experienced left pulmonary closure following treatment with anti-tuberculosis medicine for two weeks. The diameter of the left main bronchus was 2 mm as determined by electric bronchoscopy. The patient was subjected to balloon dilation three times following cryotherapy and argon coagulation. The atrophy lung lobar in the patient’s left lung stretched. Images of this case are shown in Fig. 3.

## Discussion

Endobronchial tuberculosis is one of the main causes of incurable PTB. Bronchostenosis or bronchial obstruction leads to pulmonary closure and resistance to treatment. The bronchi of the majority of patients are destroyed, obstructed and even closed as a result of PTB, which causes the pulmonary function to gradually weaken. Several patients also exhibit severe clinical symptoms or even disabilities and a number succumb to PTB. Pulmonary lobectomy is performed on patients suffering from repeated infections. If PTB is not contained appropriately prior to pulmonary lobectomy, bronchopleural fistula may occur during the procedure, followed by repeated thoracic infections that cause mortality. In the past, argon plasma coagulators and microwave coagulators or electric coagulators have been utilized for bronchostenosis. However, these electric approaches often result in the repeated growth of granulation tissue and destruction or collapse of the bronchial walls, which negatively affects the ventilation of the lung, thus compromising the effectiveness of the therapies.

Cryotherapy combined with high-pressure balloon dilation has been used to treat malignant bronchostenosis domestically and abroad (19–21). The outcomes differ each time; however, they are generally positive. The argon plasma coagulator (22) is utilized for the treatment for stenosis in the main bronchi; however, it is rarely regarded as an addition to the combined cryotherapy and balloon dilation therapy. When the argon plasma coagulator and microwave coagulator or electric coagulator are used for the electric coagulation of bronchostenosis caused by endobronchial tuberculosis (15), the granulation tissues grow repeatedly, leading to undesirable effects. We combined electric coagulation with refrigeration in this study, supplemented with balloon dilation to provide a solution to the growth of granulation tissues. Successful results were achieved. Cryotherapy decreased the local temperature to −80°C, terminated the blood supply to the granulation tissues and formed a microthrombus that obstructed the blood vessels and halted the growth of the granulation tissues. Cryotherapy also froze the cells, quickened the infiltration of inflammatory cells into the granulation tissues and the necrosis of the granulation tissues, and promoted the recovery of the intima of the bronchus. The combination of electric coagulation, cryotherapy and balloon dilation provides a solution to the growth of unwanted granulation tissues caused by PBT and bronchostenosis. Fifty-three of the 56 cases (94.6%) of bronchostenosis in our study recovered to varying degrees. The mucous membranes of the patients healed and became smooth. Symptoms, including chest distress, shortness of breath and dyspnea, also improved. Thirteen of the 15 cases (86.9%) of bronchial obstruction reopened. The majority of the pulmonary atelectatic tissues were dilated. The overall rate of recovery was 90.4%. In two of the cases, it was not possible to reopen the long segment of bronchial obstruction; therefore, the treatment was discontinued.

Several studies have shown that cryotherapy destroys cell structure, mitigates edema, suppresses the growth of tissues by reducing blood supply and activates the inflammation of tissues to hasten the death of local tissues or cells (3,4). Cryotherapy is widely utilized for prostate hyperplasia, prostatic carcinoma, cervical erosion, renal carcinoma, hepatic masses, and other diseases (23–27). Cryotherapy using a video bronchoscope also exhibits favorable effects on lung cancer (9). However, it does not demonstrate prompt efficacy since cryotherapy is a very slow-functioning procedure. Patients may undergo cryotherapy >20 times in a period of several months. Balloon dilation is administered for bronchostenosis when the condition is finally stabilized. Patients must complete the entire procedure.

Although bleeding is rarely observed during the cryotherapy of endobronchial tuberculosis or neoplasms (28), the frozen cutting of the fresh granulation tissue or neoplasm easily causes bleeding. The argon plasma coagulator is a good solution to the issue of bleeding. Frozen cutting is difficult to perform on granulomatous lesions; however, the lesions may be coagulated first by an argon plasma coagulator prior to refrigerated or frozen cutting. Given that the argon plasma coagulator also causes massive hemorrhaging when it damages the blood vessels, tissues with an abundant supply of blood or large blood vessels should be protected from the damage.

The diameter of the bronchus with stenosis and that of the usable balloon should be considered during balloon dilation to avoid massive hemorrhaging, since a large balloon may rip the bronchus. The pressure from the balloon and the injection of thrombin terminates the bleeding and so the bleeding does not cause a severe adverse outcome. None of the patients in the current study experienced massive hemorrhaging.

A large rip in the bronchus may lead to re-stenosis (29) when the bronchial stenosis heals. When balloon dilation is conducted in the left or right main bronchus at the carina of the trachea, it is important that the balloon does not block a large section of the lumen of the main bronchi. This is necessary to prevent the development of acute respiratory failure that threatens the patient’s life.

The techniques demonstrated in the current study are likely to benefit a number of patients (30). Our balloon dilation method is different from previously described methods. We dilated the bronchus continuously for 40 min at high pressure (12–14 atm), which positively affected bronchostenosis. Other balloon dilation methods dilate the bronchus for only 2 min at low pressure (<2 atm) and normally allow the immediate re-stenosis of the bronchus. Further studies on this aspect of the treatment should be conducted to add to the understanding and creation of improved techniques.

Bronchostenosis or bronchial obstruction resulting from endobronchial tuberculosis may be treated by electric coagulation, cryotherapy and balloon dilation with an electronic video bronchoscope. This sequential therapy has demonstrated the ability to heal atelectasis and avoid pulmonary lobectomy. Thus, it is considered an effective method that may be used in clinical practice.

## Figures and Tables

**Table I:** Distribution of the sites of bronchial perforation or bronchostenosis.

Sites	N (%)	Bronchostenosis	Bronchial perforation
Trachea	12 (16.9)	12	0
Left main bronchus	11 (15.5)	8	3
Right main bronchus	13 (18.3)	10	3
Right upper lobar bronchus	11 (15.5)	9	2
Right middle lobar bronchus	6 (8.5)	4	2
Right lower lobar bronchus	7 (9.9)	5	2
Left upper lobar bronchus	6 (8.5)	4	2
Left lower lobar bronchus	5 (7.0)	4	1
Total	71	56	15

Note: 15 cases demonstrated two sites of bronchostenosis.

**Table II:** Distribution of the tuberculin test (PPD) results.

Diameter of induration (mm)	0–5	6–10	11–20	>20	Total
Number of cases	21	11	15	9	56
